# Spontaneous Coronary Artery Dissection in Young Patients: A Case Series and Review of Current Management Algorithm

**DOI:** 10.7759/cureus.39393

**Published:** 2023-05-23

**Authors:** Dhan B Shrestha, Jurgen Shtembari, Kerolus Shehata, Harsha Gondi, Anish Munagala, Esteffania Villegas Santamaria, Prakash Raj Oli, Daniela Kovacs, Sandeep Khosla

**Affiliations:** 1 Department of Internal Medicine, Mount Sinai Hospital, Chicago, USA; 2 Department of Cardiology, Mount Sinai Hospital, Chicago, USA; 3 Department of Internal Medicine, Province Hospital, Surkhet, NPL

**Keywords:** coronary artery disease, obesity, coronary intervention, st-elevation myocardial infarction, spontaneous coronary artery dissection

## Abstract

Spontaneous coronary artery dissection (SCAD) is a rare but increasingly recognized non-atherosclerotic cause of acute coronary syndrome. Common risk factors for SCAD are coronary atherosclerosis, female gender, peripartum period, systemic inflammatory conditions, and connective tissue disorders. It manifests as myocardial ischemia and infarction, arrhythmia, and sudden cardiac death. We present a case series of two young men and one young female with SCAD who had chest pain and were diagnosed with SCAD-associated ST-elevation myocardial infarction.

Its diagnosis requires a high degree of clinical suspicion and its management is guided by the patient’s clinical condition and the characteristics of the lesions.

## Introduction

Spontaneous coronary artery dissection (SCAD) is rare and often underdiagnosed, especially among women with acute coronary syndrome. It leads to developing intramural hematoma compressing the arterial lumen and reducing anterograde blood flow and myocardial ischemia or infarction [1]. The overall incidence of SCAD in coronary angiographies is 0.2%. Three-quarters of the diagnosed patients are women [2]. The most commonly involved arteries are the left anterior descending (LAD) artery in women and the right coronary artery in men [2,3]. The common risk factors associated with SCAD are coronary atherosclerosis, female gender, peripartum period, connective tissue disorders such as vasculitis or antiphospholipid syndrome, hypertension, cocaine use, strenuous exercise, and hormonal therapy [1,4]. Its exact mechanism remains unknown and is presumed to result from either an intimal tear or rupture of the vasa vasorum [1,4,5]. Its clinical presentation can be highly variable and can present with chest discomfort, arm, neck, or back pain, nausea or vomiting, diaphoresis, ventricular tachycardia, and/or ventricular fibrillation [5].

SCAD can be diagnosed with coronary angiography or intracoronary imaging like intravascular ultrasound (IVUS) or optical coherence tomography (OCT). Other modalities like CT coronary angiography and cardiac magnetic resonance can add on valuable information on the diagnosis when angiography is inconclusive [1,6-8]. The management of SCAD is guided based on the clinical presentation of the patients: observation with appropriate medical therapy for stable patients with preserved coronary blood flow and revascularization strategies for unstable patients who usually present with poor coronary blood flow [1,6]. Here, we present three cases of SCAD presenting as ST-elevation myocardial infarction (STEMI).

## Case presentation

First case

**Figure 1:**
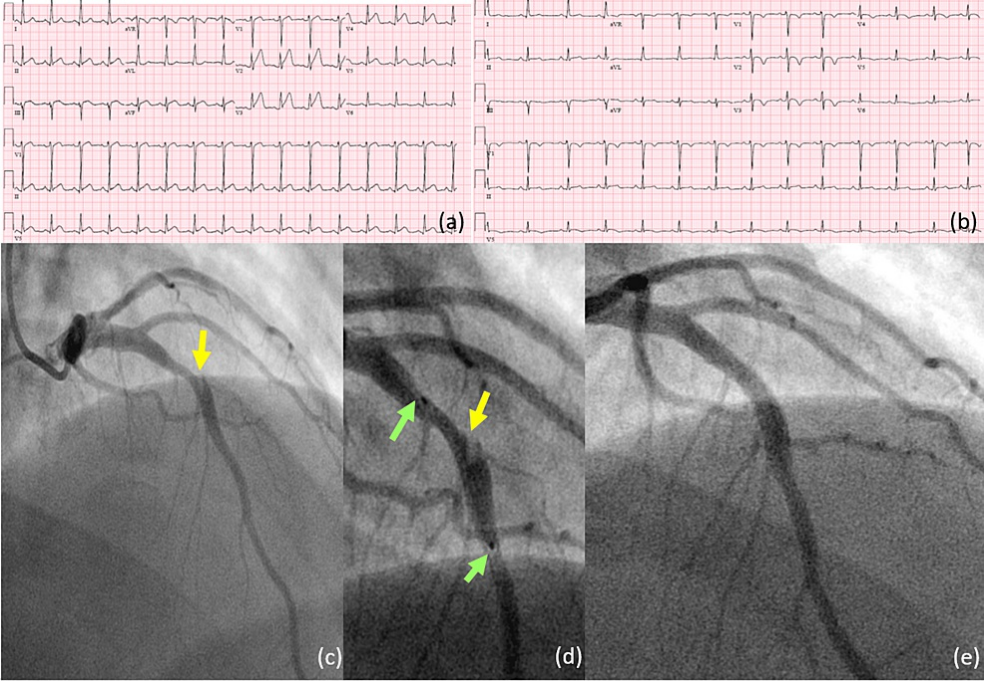
(a) ECG at presentation showing anterior ST elevations; (b) ECG post-PCI; (c) coronary angiogram showing mid-LAD dissection flap leading to 70-80% luminal stenosis; (d) coronary angiogram showing stent positioning across the dissected flap; (e) coronary angiogram showing the flow after stent deployment

Our first patient was a 27-year-old obese male (BMI 40.8 kg/m2) with no significant medical history who presented to the emergency department with sharp, severe, non-radiating midsternal chest pain that started at rest. ECG at presentation showed diffuse anterior ST elevations (Figure 1A). He had a dynamic troponin elevation and urine drug screening (UDS) was positive only for benzodiazepines. He was diagnosed with STEMI, and an emergent coronary catheterization showed LAD proximal vessel 80% stenosis with mid-LAD artery SCAD with dissection flap leading to 70-80% luminal stenosis and no evidence of thrombus or bifurcation lesion (Figure 1C). A drug-eluting stent (DES) was placed in mid-LAD (Figure 1D-1E). A transthoracic echocardiogram after the coronary intervention showed a left ventricle ejection fraction (LVEF) of 40-45%, with akinesia of the apex. He was discharged on dual antiplatelet therapy with aspirin 81 mg and clopidogrel 75 mg, lisinopril 5 mg, and metoprolol succinate 25 mg daily.

Second case

**Figure 2:**
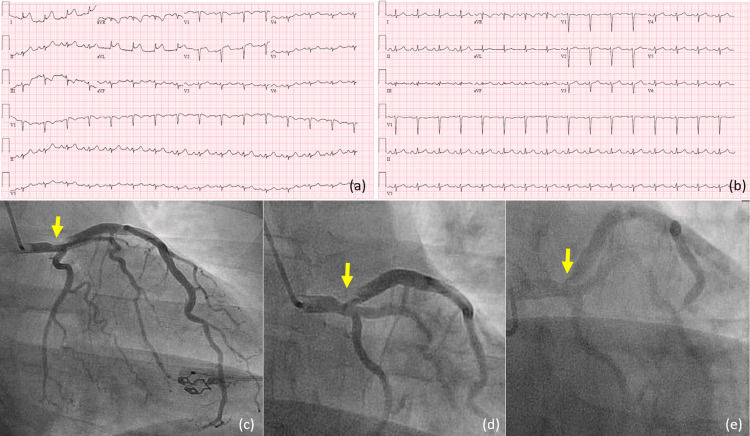
(a) ECG at presentation showing ST elevation on leads I and aVL with reciprocal changes; (b) ECG showing improvement next day; (c, d) coronary angiogram showing diffuse left main distal vessel stenosis with a SCAD extending to proximal LAD; (e) repeat coronary angiogram on the sixth day showing no expansion of dissection

The second patient was a 48-year-old morbidly obese woman (BMI 66.9 kg/m2) with hypertension and obstructive sleep apnea on continuous positive airway pressure who presented with acute, severe, nonradiating substernal chest pain that had started at rest. Her ECG showed ST elevation on leads I, aVL with reciprocal III, and aVF depressions (Figure 2A) and elevated high sensitive troponin on admission. She was diagnosed with STEMI and loaded with aspirin 325 mg, ticagrelor 180 mg, and started on a heparin infusion. Emergent cardiac catheterization showed diffuse left main distal vessel 60% stenosis with a SCAD that extended into ostial and proximal LAD with superimposed thrombus (Figure 2C-2D). This finding was reviewed with the cardiac surgery team; however, she was deemed to be a poor surgical candidate and managed with medical treatment in the cardiac care unit. The first transthoracic echocardiography revealed LVEF of 50-55% and akinesis of the corresponding myocardium. On the sixth day of her hospital stay, she underwent repeat angiography, which showed healing of SCAD with a left main distal vessel 45% stenosis, and LAD ostial lesion 60% stenosis. During the hospital stay, the patient was symptom-free, electrically, and hemodynamically stable. With angiography showing resolution of SCAD (Figure 2E), no further intervention was recommended, and the patient was discharged on dual antiplatelet therapy with aspirin 81 mg and ticagrelor 90 mg twice daily, lisinopril 20 mg, metoprolol tartrate 100 mg twice daily, isosorbide mononitrate 30 mg, and close follow-up in our cardiology clinic.

Third case

**Figure 3:**
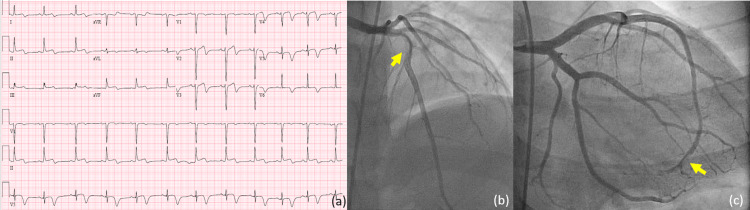
(a) ECG at presentation showing type A Wellens sign; (b, c) diffuse, mid-LAD lesion with 60% de novo spontaneous dissection with distal vessel 85% lesion

Our third patient was a 19-year-old morbidly obese (BMI 47.4 Kg/m2) male evaluated for a pressure-like sensation in his throat and paresthesia in the left arm, associated with nausea and vomiting, and his symptoms lasted 30 minutes. His family history was significant for MI at a young age. The first EKG showed discrete ST elevations with associated new T wave inversions in the inferior leads (Figure 3A). High-sensitive troponins were significantly elevated on admission, and UDS tested positive only for cannabinoids. Emergent coronary angiogram showed a diffuse, mid-LAD lesion with 60% de novo spontaneous dissection with distal vessel 85% lesion (Figure 2B-2C). The patient was loaded with aspirin 325 mg and ticagrelor 180 mg. Low-molecular-weight heparin with enoxaparin 140 mg twice daily was used for anticoagulation. Transthoracic echocardiography showed a preserved systolic function with an LVEF of 55-60% and no regional wall motion abnormalities. A repeat coronary angiogram after 48 hours showed a resolving diffuse mid-vessel lesion with 40% de novo spontaneous dissection and a diffuse distal vessel with 20% de novo spontaneous dissection (SCAD-2A). The lesion had an improved angiographic appearance compared to a prior coronary angiogram and excellent TIMI 3 flow. He was discharged on dual antiplatelet therapy with aspirin 81 mg, ticagrelor 90 mg twice daily, and metoprolol succinate 12.5 mg daily. He was scheduled for a relook coronarography one month after discharge.

Table 1 shows the summary of all three cases.

**Table 1:** Showing summary of all three cases BMI: body mass index; EKG: electrocardiography; HS-troponin-I: high-sensitivity troponin I; PCI: percutaneous coronary intervention; DES: drug-eluting stent; LAD: left anterior descending; SCAD: spontaneous coronary artery dissection; LVEF: left ventricle ejection fraction; DAPT: dual antiplatelet therapy, NSTEMI: non-ST-elevation myocardial infarction

Characters	1^st ^case	2^nd^ case	3^rd^ case
Age	27 years old	48 years old	19 years old
Sex	Male	Female	Male
BMI	40.8 kg/m^2^	66.9 kg/m^2^	47.4 kg/m^2^
Troponin-I/HS-troponin-I	<0.03>0.63>5.20>15.25>17.53 ng/dl	194>7327>6986 pg/ml	0.2 >1.2>18 ng/dl
Urine drug screen	Positive for benzodiazepines	-	Positive for marijuana
EKG	Anterior ST elevations	ST elevation on leads I and aVL	Anterolateral T wave inversions
PCI	LAD proximal vessel 80% stenosis with mid-LAD SCAD with dissection flap leading to 70-80% luminal stenosis	Left main distal vessel diffuse, 60% stenosis with a SCAD with extension into ostial and proximal LAD with superimposed thrombus	Diffuse, mid-LAD lesion with 60% de novo spontaneous dissection with the distal vessel, 85% thrombosis
Intervention	DES placed to mid-LAD	Medical management	Medical management
ECHO	LVEF of 40-45%, with akinesia of apex	LVEF of 50-55%, akinesis of mid-anteroseptal, mid-inferoseptal, apical septal, and apical myocardium	LVEF of 55-60%, no regional wall motion abnormalities
Relook coronary angiography		Left main distal vessel with 45% stenosis and LAD ostial lesion with 60% stenosis and no SCAD noted	Diffuse mid-LAD lesion with 40% de novo spontaneous dissection with the diffuse distal vessel and 20% de novo spontaneous dissection (SCAD-2A)
Further management	DAPT (aspirin 81mg and clopidogrel 75mg), metoprolol succinate 25mg, and lisinopril 5mg	DAPT (ticagrelor 90mg twice daily and aspirin 81 mg daily), metoprolol tartrate 100mg twice daily, and lisinopril 20mg	DAPT (ticagrelor 90mg twice daily and aspirin 81 mg daily), metoprolol succinate 12.5 mg daily

## Discussion

SCAD was first reported by Pretty HC in a 42-year-old woman at autopsy in 1931. After that, it was well studied, but its true prevalence was underestimated and had varying presentations [1,9,10]. The reported incidence of SCAD varies from 0.07 to 4.0%, [1,5-7,10,11]. It mainly occurs in women aged 45-53 with high-risk factors like coronary atherosclerosis, connective tissue disorder, systemic inflammatory disorders, postpartum period, fibromuscular dysplasia, and low-risk factors like hypertension, diabetes, and dyslipidemia [1,4,8,10,12].

Overweight and obesity were reported as risk factors for SCAD in a review study by Neubeck et al. in 2022. The incidence of overweight ranged from 22.2% to 27%, and the incidence of obesity ranged from 11.8 % to 33.3% of participants in six studies that were reviewed [13]. Other common risk factors in this review were fibromuscular dysplasia, migraine, and hypertension.

SCAD mainly involves mid-distal areas of the LAD coronary artery, especially in women, and the right coronary artery, especially in men [2,3,6,8-10]. We presented three cases of STEMI-associated SCAD. Our first patient had a mid-LAD artery SCAD, the second patient had SCAD involving distal areas of the LAD with its ostial extension and superimposed thrombus in its proximal part, and the third patient had mid-distal vessel SCAD. All three cases presented have morbid obesity, one of the risk factors associated with SCAD.

Coronary angiography is the first line of investigation with some limitations in precisely localizing the dissection entry point and identifying the true and false lumen. When coronary angiography can’t make the diagnosis, intracoronary imaging like IVUS and OCT can be used, as these imaging modalities give a more detailed analysis of the lesion [1,4,5,7,12,14]. However, intracoronary imaging may not be feasible in each case, particularly in those with severe tortuosity or when it involves more distal small-caliber arteries. In patients with diagnostic uncertainty, follow-up coronary angiography, either invasive or computed tomography angiogram, can confirm SCAD diagnosis if it shows the healed SCAD with normal anatomy [8]. In our case, SCAD was diagnosed with coronary angiography with diagnostic accuracy without requiring further intracoronary imaging modalities.

The key to its successful management is early diagnosis and treatment, as most of them heal spontaneously with good long-term outcomes if they overcome the initial event [1,7]. There are three treatment strategies: medical treatment, stent placement, and coronary artery bypass graft (CABG), and the treatment modality is guided by the patient’s clinical presentation, the hemodynamic state, and the character of dissection [7,11]. The 2021 ACC/AHA/SCAI guidelines for coronary artery revascularization refer to the treatment of SCAD as challenging and guided by evidence drawn from observational studies in which patients were managed conservatively with a lack of evidence from randomized trials. Revascularization procedures are recommended for SCAD patients with hemodynamic instability or ongoing ischemia despite conservative therapy and deter the routine use of revascularization due to its more harmful effects than benefits [15].

Medical treatment involves antiplatelet agents such as aspirin, glycoprotein IIb/IIIA receptor antagonist, receptor P2Y12 inhibitors, anticoagulation therapy, beta-blockers, and ACEIs [7,11]. Fibrinolytic therapy in SCAD has a possible double-edged effect [11]. Medical treatment is effective but has a weak scientific evidence base due to a lack of randomized control trial studies [5]. Medical treatment might have better recovery among ACS-associated SCAD patients [1,6,11]. Medical management of SCAD with the healing of the lesion has been documented in a case series of nine patients by Ghani et al., four cases had a stable disease involving mid LAD, and LCx were managed and healed with medical therapy without complications [5]. Other case reports such as the cases by Regragui et al. [14] or Abdullah et al. [16] presented patients with SCAD involving distal LAD with ostial extension and LCx or proximal to distal RCA SCAD, respectively, that were successfully managed with medical treatment with final healing of the lesion.

Stent placement or CABG is considered for patients with more proximal dissection, hemodynamic instability, dynamic ST-segment changes, and ventricular arrhythmias [4,12]. PCI is associated with the risk of dissection extension, guidewire passage into the false lumen, and major side branch restriction or blockade by hematoma [1,14]. In the long term, there is a risk of stent malposition and an increased risk of late stent thrombosis with intramural hematoma resorption and healing, especially after cessation of dual antiplatelet therapy [1]. CABG has no significant long-term protection from recurrent SCAD; thus, it should be considered for those with persistent or recurrent ischemic pain after failed PCI and multivessel or left coronary arteries artery SCAD [11,14,17]. CABG is also associated with a risk of connecting false lumen, which mandates extra caution for proper identification of the lesion [5].

Our patients reflect the current medical knowledge about SCAD. Revascularization was elected for the first patient, and medical management was preferred in the other two cases. Repeat coronary angiography revealed the resolving severity of the lesions providing evidence of healing. Challenging cases can be seen in the case of our 48-year-old female patient with an ostial LAD lesion. She was a poor candidate for revascularization therapy. She did well with medical management, and her lesion improved as it was later seen in the repeat angiography.

Lastly, this is a series of three cases and the findings that we report cannot be generalized. Our experience is limited and our impression of obesity and SCAD relationship is biased by the restricted number of patients. We encourage larger studies that would help build the level of evidence on the management of SCAD.

## Conclusions

Despite its low incidence, SCAD is an important cause of ACS that is mainly seen in young individuals. Its diagnosis requires a high degree of clinical suspicion, and coronary angiography is the most commonly used imaging modality to make a diagnosis. Its management is guided by the patient’s status and the lesion’s characteristics. SCAD management is guided by low-level evidence and requires further studies that will help improve clinical outcomes.
